# Pasteurization Versus Purity1Read before the meeting of the Pennsylvania State Veterinary Medical Association, Philadelphia, March 8, 1898.

**Published:** 1898-04

**Authors:** H. B. Felton

**Affiliations:** Philadelphia, Pa.


### PASTEURIZATION VERSUS PURITY.[1]

By H. B. Felton, M.D.,
PHILADELPHIA, PA.

The above title has been suggested by a paper read recently by Dr. John I. Carter before the Avondale Institute, in which he condemns the pasteurization of milk, claiming that by this process the life-principle is taken out of the milk, and it is rendered unwholesome and innutritious.

Dr. Carter rightfully contends for "an absolutely wholesome milk, produced by a healthy, vigorous cow, fed on sweet, wholesome food, kept in untainted environments, milked in a cleanly manner, and the product kept from after-contamination." Such a milk is indeed an ideal product, and in no need of pasteurization. The nearest approach to it at the present time is the milk produced upon the "Walker-Gordon" dairy-farms, where every precaution known to modern science is taken to prevent contamination. Such a milk may be regarded as above suspicion; but, unfortunately, the amount of it produced as compared with the total supply is but as a drop in the bucket.

It has occurred to me that in the present condition of our milk-supply it is unwise to condemn pasteurization, and that it is the most valuable means we possess of safeguarding one of our most important food-products.

Milk is one of the most favorable mediums for the growth of bacteria. As produced and sold in the ordinary way it literally swarms with them. Samples taken at random from the milk sup-

[1] Read before the meeting of the Pennsylvania State Veterinary Medical Association, Philadelphia, March 8, 1898.

plied to any of our large cities will show anywhere from 500,000 to 1,500,000 bacteria per cubic centimetre. A large number of the bacteria found in milk are such as cause the usual acid fermentation which occurs when milk turns sour; but there are many species which ought to be excluded, arising from mouldy hay, straw, or fodder, partially decayed roots, and the natural decay of the woodwork of the barn and adjoining buildings. Many of these bacteria cause alkaline fermentation and other abnormal conditions of milk.

The question is often asked, Why are we not all destroyed by the countless numbers of microbes that are in the air we breathe, in the water we drink, and the food which we swallow? Dr. H. Beauregard, an eminent physician of Paris, in an article on "Microbes and Man" in the *Revue Pedagogique*, a translation of which appears in the *Literary Digest* of January 8, 1898, has shown that the human body is perfectly organized to resist the different phases of the attacks of the microbes, and has also shown how we may succumb to them. Before reaching us they have already encountered conditions which put them in a certain measure in a state of inferiority. The oxygen of the air and light are agents which injure their vitality. From this fact arises the importance of hygiene. Having reached the skin, microbes find an efficacious barrier in the cells of the epidermis of which those on the surface are horny and in a continual state of desquamation. This may be called the physical defence of the epidermis. The skin also contains glands producing sweat and oily matter. These substances are eminently unfavorable for keeping up life in microbes. Should the microbes penetrate into the glands themselves, they are borne out upon the current of gland-secretion when the gland is excited into action. Those microbes which enter the mouth and nostrils find a membrane lining these composed of cells not unlike the cells of the epidermis, and this membrane is constantly moistened with liquids which are not at all favorable to the development of the assailant. If the microbes manage to get into the œsophagus, and so reach the stomach, they find there conditions which are not good for their health in the shape of chlorohydric, lactic, and other acids. According to Escherich, the bacillus lactis ærogenes is found normally in the stomach, and is responsible for the conversion of milk-sugar into lactic acid, which is a powerful germicide for the other forms of bacteria. Many microbes are absolutely incapable of getting through the stomach and penetrating into the intestines, for they have been so battered and knocked about, and their vitality has been so much lowered by their troubles on the road, that they end by being destroyed and even digested in the stomach. It has been proved, however, that mucous surfaces are not always an obstacle to the penetration of the microbe even when these surfaces are intact. Suppose the microbes manage to penetrate the tissues, then they meet with new obstacles. They find, in the first place, what are called phagocytes that is, cells which are eaters. These elements of the lymph show surprising activity, swallowing the microbes and digesting them. These phagocytes are most abundant at threatened points. If, in spite of the phagocytes, the microbes get into the blood, they have not won the battle yet. The serum of the blood has microbe-killing properties. The oxygen that is carried into the blood disagrees with many of the microbes, as carbonic acid does with others, and thus it is that the blood is rarely invaded with microbes in the course of the maladies they engender. If the microbes take up their residence in the heart of the organs, even there they meet with elements of resistance which are often efficacious, such as defensive proteins and other antitoxic substances produced by these organs.

When, however, the general functions of the system are troubled either hereditarily or by reason of an acquired abnormal state, such as gout, diabetes, visceral, pulmonary, or hepatic inflammation, the conditions of resistance are changed, for these, by vitiating the regular functions of the organs, affect the vitality of the tissues and particularly the phagocytic elements. The microbes are destroyed in much smaller quantities, and they no longer find antitoxic products which ought normally to oppose their development and neutralize the effect of their own poisons. In a word, they find a field of culture in which they cannot fail to flourish and multiply. The consequences are immediate, the infection of the tissues begins, and the poisons produced by the microbes are spread throughout the organism.

Such is the mechanism of the origin of diseases called infectious. From all this it is plain that everything which enfeebles our vitality is a dangerous condition and exposes to invasion. The most varied influences can come into play to create in us a condition of inferiority which will oblige us to surrender to our foe. Privations, great fatigue, the ingestion into our systems of toxic substances, intoxication by lead or alcohol, atmospheric conditions, excessive heat, intense cold, are so many elements which must be reckoned with.

There is no warrant, then, for neglecting microbes and considering them an enemy of slight importance. It would be folly to think that we may fold our arms and trust to our natural powers of resistance. On the contrary, we should always keep in mind that we have in microbes terrible adversaries, always on the alert to surprise us, and against which we are bound to maintain as intact as possible the natural defences with which our organism can oppose them. We believe one of our most valuable allies to be pasteurization. In our opinion Pasteur has done no more valuable service for mankind than in giving us the process which bears his name. When we think of the vast number of infants deprived of the mother's milk, which nature intended them to imbibe, and dependent upon the cow for their source of supply; when we consider that the natural powers of resistance of the infant are far below those of the adult, and that by reason of the ennervating heat of summer, or the cold of winter, or bad hygienic surroundings, these powers are still further weakened, we see how important it is that they should be supplied with a food free from germs. It has been proved that milk has been the medium through which tuberculosis, typhoid fever, scarlet fever, and diphtheria have been contracted. The colon bacillus is held responsible for producing ileo-colitis, or the ordinary summer-complaint of children. Milk rich in bacteria is regarded by the highest authorities as being difficult of digestion for children, and causing food-vomiting, dyspepsia, mycotic diarrhœa and ileo-colitis.

In the absence of a systematic inspection of milk at the dairies by trained veterinarians, how can we know that the milk-supply will be free from these germs? We do know that pasteurization will destroy these germs. By pasteurization we mean heating the milk to 167° F. and keeping it at that temperature for forty minutes by means of live steam. This has been found to be the best method, and is that practised in the Walker-Gordon laboratories. The highest thermal death-point of all germs ordinarily found in milk is 157° F., so that a safe margin is allowed. It is important that the milk be rapidly cooled after this process, in order that bacteria may not develop during the time of cooling. It is most conveniently carried on in vessels containing not more than half a pint, which must be tightly stoppered and are not to be opened until used.

Milk pasteurized in this way does not lose its distinctive taste or odor, nor is its nutritive value or digestibility impaired in any way. It has been claimed that by this process the life-principle is destroyed. We do not believe that any life is destroyed except that which is harmful by its presence. The coagulation of the proteids does not begin until 171° F. is reached, when changes take place which render the milk more difficult of digestion.

The Walker-Gordon laboratories are supplying large numbers of infants and children in all of our large cities with milk that has been pasteurized. Although they furnish milk comparatively free from germs, it is deemed best as a matter of precaution to subject it to this process. Physicians are getting results by means of this scientific feeding rendered possible by these milk-laboratories that they have never attained before. We believe that the pasteurizing-kettle will become a rival of the tea-kettle when its virtues are more widely known. Science has given us a clear light upon this subject. Let us not go back to the darkness of the past.

